# Impact of moderate-to-late preterm birth on neurodevelopmental outcomes in young children: Results from retrospective longitudinal follow-up with nationally representative data

**DOI:** 10.1371/journal.pone.0294435

**Published:** 2023-11-16

**Authors:** Sangmi Lee, Yuri Han, Min Kyung Lim, Hun Jae Lee

**Affiliations:** 1 Department of Nursing, College of Nursing, Dongyang University, Yeongju-si, Gyeongsangbuk-do, Republic of Korea; 2 Department of Social and Preventive Medicine, College of Medicine, Inha University, Incheon, Republic of Korea; Xi’an Jiaotong University, CHINA

## Abstract

This study investigated the relationship between moderate-to-late preterm (MLPT) birth and the risk of neurodevelopmental impairments (NIs) in young children compared with the risks associated with very preterm (VPT) and full-term (FT) birth based on nationally representative large-scale population data. Retrospective follow-up was conducted over 71 months for 738,733 children who were born and participated in the Korean National Health Screening Program for Infants and Children (NHSPIC) between 2011 and 2013. Using a data linkage between the NHSPIC and Korean healthcare claim information, data on birth year, sex, delivery type, birth weight, growth abnormality, gestational age, breastfeeding history, maternal age, NIs, multiple gestation, preterm labor, premature rupture of membranes (PROM), gestational diabetes, gestational hypertension, smoking during pregnancy, and socioeconomic status were collected and included in the final analysis. Cox proportional hazards models were applied to identify the impact of gestational age on NI risk, with all variables adjusted as appropriate. Overall, 0.9% and 3.8% rates of VPT and MLPT births were identified, respectively. NI incidence was highest among VPT children (34.7%), followed by MLPT (23.9%) and FT (18.2%) children. Both VPT (hazard ratio [HR], 1.45; 95% confidence interval [CI], 1.03 to 2.05) and MLPT (HR, 1.21; 95% CI, 1.04 to 1.41) births were associated with increased NI risk. Low birth weight, PROM, and smoking during pregnancy were also associated with increased NI risk, while longer breastfeeding and higher socioeconomic status were associated with decreased risk. Special attention must be given to NIs for both VPT and MLPT children.

## Introduction

The rate of preterm (PT) birth has steadily risen over the past 20 years, reaching approximately 11% of annual global births worldwide [1]. PT birth is a major healthcare problem, as it is the leading cause of child mortality in countries with relatively low mortality due to infectious diseases. Children born PT are particularly susceptible to cognitive, motor, behavioral, and academic impairment and are especially vulnerable to developmental complications such as brain dysfunction [2].PT can be subclassified as extremely preterm (EPT) (<28 weeks), very preterm (VPT) (28 to 32 weeks), and moderate-to-late preterm (MLPT) (32 to 37 weeks) [3]. In most studies on neurodevelopmental outcomes, the focus has been on EPT and VPT births, as they have higher risk and greater clinical severity related to the relatively short gestational age involved [2, 4–6]. However, some previous studies have also suggested that relative to full-term (FT) birth, MLPT birth carries an increased risk of brain growth and maturation delay [7] as well as brain injuries, such as intraventricular hemorrhage and white matter injury [7, 8]. Furthermore, children born MLPT have been found to have higher incidence rates of cerebral palsy; more frequent delayed development of cognitive, fine motor, and gross motor skills; communication skill deficits; and delayed social skills [9–11].

However, studies of the impacts of MLPT birth on neurodevelopmental outcomes are still lacking, and the results of most previous studies are mainly concerned with the short-term effects within 24 months after MLPT birth, although the reduced brain volume and brain injury of children born MLPT can affect their neurodevelopment at 2 years and even up to 9 years [12, 13], and the neurodevelopmental delay at the age of 2 years for children born MLPT can persist into preschool age [14]. Even a study with a relatively long follow-up period included only a small number of children born MLPT admitted to a hospital, without any comparison group [15]. Another study included a community-based large-scale dataset of children aged 1–59 months born MLPT, but it was limited by its cross-sectional design [16].

Therefore, a large population-based long-term follow-up study appears necessary to overcome limitations due to the low incidence of MLPT-related diseases, as well as to explore the long-term impact of MLPT birth on children’s neurodevelopmental outcomes.

In the Republic of Korea, the percentage of PT births has more than tripled from 2.55% in 1995 to 9.12% in 2021 [17], and the vast majority (approximately 90%) of PT births are MLPT [17]. However, very few studies have been published regarding the impact of MLPT birth on neurodevelopmental outcomes in children.

Therefore, the current study was conducted to investigate this impact based on nationally representative large-scale data from the National Health Screening Program for Infants and Children (NHSPIC) and healthcare claim data from the National Health Insurance Sharing Service (NHISS) in Korea using a retrospective cohort design.

## Methods

### Participants and data collection

This study was conducted using data from 2011 to 2018 that were obtained from the NHPIC database in July 2021. The NHSPIC is a national health screening service that has been provided free of charge to infants and children under 6 years of age since November 2007 by the Ministry of Health and Welfare of Korea to support the health management of young children. The NHSPIC consists of self-reported questionnaires, anthropometric measurements, and developmental screening tests, and it involves repeatedly gathering health information on these measures at several intervals (4–6 months, 9–12 months, 18–24 months, 30–36 months, 42–48 months, 54–60 months, and 66–71 months after birth). To ensure the accuracy and reliability of health screening results, the NHSPIC is conducted by trained doctors at designated examination institutions in accordance with the Korea Disease Control and Prevention Agency’s manual [18], and the screening rate is high (87.1% as of 2021) [19].

Children who were born and participated in the NHSPIC between 2011 and 2013 were included in the current study as the baseline population; their data were linked to maternal data from the Korean NHISS, and those data were also linked to NHISS healthcare claim data, which contains information on gestational age and type of gestation based on the International Classification of Diseases, Tenth Revision (ICD-10). All data in this study were provided through the review process after submitting a data request with a study proposal and notification of IRB approval to NHISS of the National Health Insurance Service (https://nhiss.nhis.or.kr/bd/ay/bdaya001iv.do).

**Fig 1:**
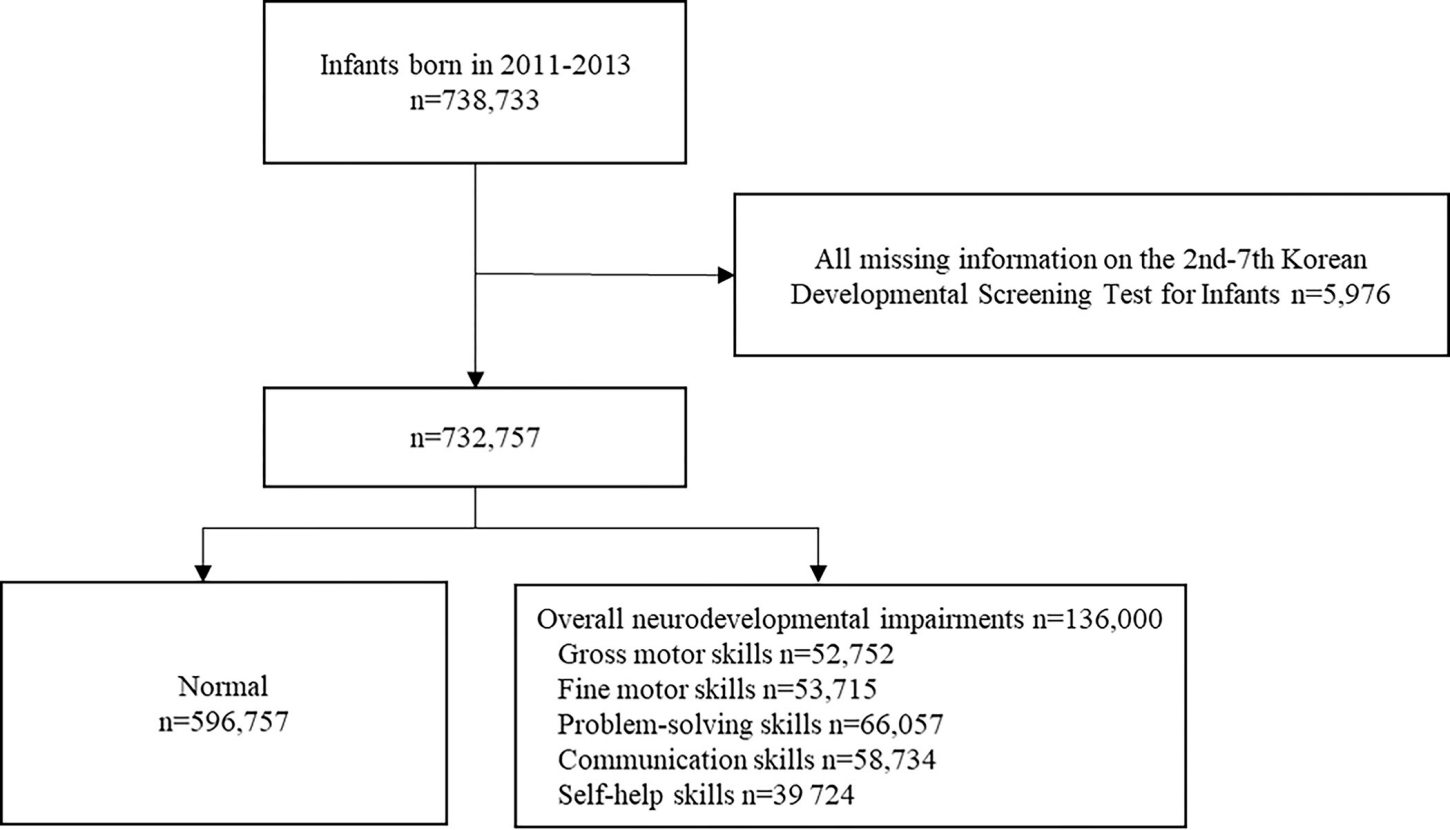
Schematic flow of study participants.

In the final analysis, 732,757 of the 738,733 children were included after exclusion of 5,976 children who were missing information from the second to seventh developmental screenings (Fig 1).

### Ethical considerations

Review exemption was obtained from the Institutional Review Board (IRB No.: 1041495-202005-HR-01-01/1041495-202108-HR-03-01) of the institution to which the researcher belongs before proceeding with the current study, because this study was conducted using data from the NHSPIC and healthcare claims data from 2011 to 2018 provided by the NHISS in the Republic of Korea. We were provided suitable data for the purpose of this study, with no identifiable personal information, after the NIHSS granted approval for data provision. As the acquired data can only be accessed from the NHISS Analysis Center, after study data construction was completed, the researcher visited the Analysis Center on a periodic basis from September 2021 to August 2022 to conduct data analysis.

### Variables

From the NHSPIC data, information on birth year, sex, mother’s reported birth weight, weight and height measured at each screening, and mother’s reported history of breastfeeding were collected.

Birth weight was classified as <2.5 kg, 2.5 to 3.9 kg, or ≥4.0 kg in accordance with World Health Organization guidelines on low birth weight and overweight for children [20]. Weight and height data measured at the fourth screening were classified as normal (both normal weight and height) or abnormal (either abnormal weight or abnormal height of at least 3 or fewer than −3 standard deviations) in accordance with the growth curve suggested by the Korean National Growth Charts for Children and Adolescents in 2017 [21]. Breastfeeding history was classified as none, 6 months, or 1 year based on the duration of exclusive or partial breastfeeding. The Korean Ages and Stages Questionnaires (K-ASQ) and Korean Developmental Screening Test for Infants and Children (K-DST) were used before and after September 2014, respectively, to screen for neurodevelopmental impairments (NIs) including impairments in gross motor skills, fine motor skills, problem-solving, communication, and self-help. If the results of the K-ASQ and K-DST indicated “follow-up needed” (or “further testing needed”) at least once during the screening follow-up, the child was classified as having impairments [22]. Additionally, maternal health behavior (e.g., smoking during pregnancy) was investigated using national medical checkup data for adults.

Maternal age was grouped as <40 years or ≥40 years, and socioeconomic status was classified by National Health Insurance Premium quintiles.

Using the NHISS healthcare claim data, the type of gestation was classified as single or multiple based on the presence or absence of records with the ICD-10 codes O30.0-O30.2 or O30.8-O30.9. Gestational age was grouped into FT or PT, and PT was then subcategorized into MLPT (32 weeks to fewer than 36 weeks based on the ICD-10 codes P07.31 and P07.32) and VPT (28 weeks to fewer than 32 weeks based on the ICD-10 code P07.30). Obstetric factors, such as cesarean section delivery (ICD-10 codes O82 or O82.0-O82.2 or O82.8-O82.9 or O84.2, or fee-for-service codes R4507- R4510, or R5001-R5002 or R4514 or R4516- R4520), gestational diabetes (ICD-10 code O24.4), gestational hypertension (ICD-10 code O13), preterm labor (ICD-10 codes O60.0-O60.3), and premature rupture of membranes (PROM) (ICD-10 codes O42.0-O42.2 or O42.9) were classified as “yes” or “no” based on the presence or absence of records with the ICD-10 codes or fee-for-service codes corresponding to that.

### Statistical analysis

The number of person‐months was calculated from the date of first developmental screening to the date of any NI screening, follow-up loss, or last follow‐up, whichever came first. The frequency distributions of the general characteristics of study participants were analyzed and presented by gestational group and sex. Multivariable Cox proportional hazards models were used to identify the impact of gestational age on the risk of NI after adjusting for birth year, sex, cesarean section delivery, birth weight, growth abnormality, history of breastfeeding, maternal age, socioeconomic status, type of gestation, preterm labor, PROM, gestational diabetes, gestational hypertension, and smoking during pregnancy. The results were presented as hazard ratios (HRs) and 95% confidence intervals (CIs). A p-value of less than 0.05 was considered to indicate statistical significance. All statistical analyses were performed using SAS 9.4 (SAS Institute, Cary, NC, USA).

## Results

The mean follow-up period of the 732,757 children was 3.1 years, and 136,000 children were identified as having NIs (impairments of gross motor skills, 52,752; impairments of fine motor skills, 53,715; impairments of problem-solving skills, 66,057; impairments of communication skills, 58,734; or impairments of self-help skills, 39,724) (Fig 1).

Among 732,757 children born between 2011 and 2013, the number of children born PT was 38,660 (5.3%), including 6,460 children (0.9%) born VPT and 28,195 (3.8%) born MLPT. The rate of PT birth increased with increasing birth year, male sex, underweight (<2.5 kg) status, cesarean section delivery, shorter duration of breastfeeding, maternal age of 40 years or older, lower socioeconomic status, multiple gestation, preterm labor, PROM, gestational diabetes, gestational hypertension, and maternal smoking during pregnancy. However, it did not differ by growth abnormality. These patterns were similar in children born MLPT and VPT, except for sex and socioeconomic status in the latter (Table 1).

**Table 1:** General characteristics of study subjects by gestational age group.

	Total (n = 732,757)	FT (n = 694,409)	PT					
			PT (n = 38,348)	p value	VPT (n = 6,460)	p value	MLPT (n = 28,195)	p value
**Children variables**								
** Birth year**								
2011	246,499	234,418 (95.1)	12,081 (4.9)	< .0001	2,028 (0.9)	< .0001	8,889 (3.7)	< .0001
2012	254,547	241,369 (94.8)	13,178 (5.2)		2,199 (0.9)		9,699 (3.9)	
2013	231,711	218,622 (94.4)	13,089 (5.7)		2,233 (1.0)		9,607 (4.2)	
** Sex**								
Boy	388,462	367,518 (94.6)	20,944 (5.4)	< .0001	3,480 (0.9)	.1300	15,446 (4.0)	< .0001
Girl	344,295	326,891 (95.0)	17,404 (5.1)		2,980 (0.9)		12,749 (3.8)	
** Birth weight (kg)** ^**a**^ ^**)**^								
Normal (≥2.5, <4.0)	649,485	638,365 (98.3)	11,120 (1.7)	< .0001	501 (0.1)	< .0001	9,419 (1.5)	< .0001
Low (<2.5)	61,451	34,321 (55.9)	27,130 (44.2)		5,940 (14.8)		18,714 (35.3)	
High (≥4.0)	21,765	21,672 (99.6)	93 (0.4)		17 (0.1)		59 (0.3)	
** Cesarean section delivery**								
No	456,660	438,745 (96.1)	17,915 (3.9)	< .0001	2,786 (0.6)	< .0001	13,318 (2.9)	< .0001
Yes	276,097	255,664 (92.6)	20,433 (7.4)		3,674 (1.3)		14,877 (5.4)	
** Abnormality of growth** ^**b**^ ^**)**^								
Normal	582,407	552,948 (94.9)	29,459 (5.1)	.771	4,720 (0.9)	.1462	21,927 (3.8)	.7035
Abnormal	3,132	2,970 (94.8)	162 (5.2)		18 (0.6)		122 (4.0)	
** History of breastfeeding**								
No	148,274	138,797 (93.6)	9,477 (6.4)	< .0001	981 (0.7)	< .0001	7,608 (5.2)	< .0001
6month	111,358	107,633 (96.7)	3,725 (3.4)		348 (0.3)		3,045 (2.8)	
1year	132,706	129,567 (97.6)	3,139 (2.4)		238 (0.2)		2,615 (2.0)	
**Maternal variables**								
** Age (years)**								
<40	710,878	674,357 (94.9)	36,521 (5.1)	< .0001	6,153 (0.9)	< .0001	26,843 (3.8)	< .0001
≥40	21,879	20,052 (91.7)	1,827 (8.4)		307 (1.5)		1,352 (6.3)	
** Socioeconomic status** ^**c**^ ^**)**^								
1	95,424	90,331 (94.7)	5,093 (5.3)	< .0001	900 (1.0)	.3796	3,712 (4.0)	.0007
2	125,079	118,755 (94.9)	6,324 (5.1)		1,091 (0.9)		4,680 (3.8)	
3	192,121	182,524 (95.0)	9,597 (5.0)		1,655 (0.9)		7,055 (3.7)	
4	205,872	194,848 (94.7)	11,024 (5.4)		1,790 (0.9)		8,167 (4.0)	
5	110,043	103,949 (94.5)	6,094 (5.5)		987 (0.9)		4,428 (4.1)	
** Multiple gestation**								
No	731,071	693,715 (94.9)	37,356 (5.1)	< .0001	6,228 (0.9)	< .0001	27,527 (3.8)	< .0001
Yes	1,686	694 (41.2)	992 (58.8)		232 (25.1)		668 (49.1)	
** Preterm labor**								
No	664,593	647,455 (97.4)	17,138 (2.6)	< .0001	2,151 (0.3)	< .0001	12,997 (2.0)	< .0001
Yes	68,164	46,954 (68.9)	21,210 (31.1)		4,309 (6.3)		15,198 (22.3)	
** PROM**								
No	710,791	683,423 (96.2)	27,368 (3.8)	< .0001	4,087 (0.6)	< .0001	20,258 (2.9)	< .0001
Yes	21,966	10,986 (50.0)	10,980 (50.0)		2,373 (10.8)		7,937 (36.1)	
** Gestational diabetes**								
No	654,607	621,662 (95.0)	32,945 (5.0)	< .0001	5,533 (0.8)	< .0001	24,222 (3.7)	< .0001
Yes	78’150	72,747 (93.1)	5,403 (6.9)		927 (1.2)		3,973 (5.1)	
** Gestational hypertension**								
No	723,103	686,678 (95.0)	36,425 (5.0)	< .0001	6,112 (0.8)	< .0001	26,740 (3.7)	< .0001
Yes	9,654	7,731 (80.1)	1,923 (19.9)		348 (3.6)		1,455 (15.1)	
** Smoking during pregnancy**								
No	60,703	57,703 (95.1)	3,000 (4.9)	0.0112	489 (0.8)	0.050	2,178 (3.6)	0.051
Yes	515	477 (92.6)	38 (7.4)		10 (1.9)		24 (4.7)	

Note: P-values were obtained from the chi-square test for categorical variables. VPT: Gestational ages of less than 32 weeks. MLPT: Gestational ages of 32 weeks to less than 36 weeks.

Abbreviations: FT, Full term; PT, Preterm; VPT, Very-preterm; MLPT, Moderate-to-late preterm; PROM, Premature rupture of membranes.

^a^Classified by the criteria for normal, low, and high birth weight according to the WHO recommendation [20].

^b^Classified as normal (having both normal weight and height) or abnormal (either abnormal weight or abnormal height) based on the weight and height data measured at fourth screening following the growth curve suggested by Korean National Growth Charts for Children and Adolescents [21].

^c^National Health Insurance Premium quintiles (highest to lowest from 5 to 1).

The incidence of NI as indicated by K-ASQ and K-DST was higher in children born MLPT (23.9%) than in children born FT (18.2%), although it was highest in children born VPT. This result was observed regardless of sex. Children born MLPT as well as those born VPT showed higher incidence rates of NI in both age-adjusted and full models relative to children born FT. Although the HR of children born MLPT was lower than that of children born VPT, both groups were significantly associated with NI, at 1.45 (95% CI, 1.03 to 2.05) and 1.21 (95% CI, 1.04 to 1.41), respectively (Table 2).

**Table 2:** HRs and 95% CIs for overall neurodevelopmental impairment by sex.

	Total				Boy				Girl			
	Total	Overall NI			Total	Overall NI			Total	Overall NI		
		Impairments (n, %)	HR (95%CI)^a^^)^	HR (95%CI)^b^^)^		Impairments (n, %)	HR (95%CI)^a^^)^	HR (95%CI)^b^^)^		Impairments (n, %)	HR (95%CI)^a^^)^	HR (95%CI)^b^^)^
**Children variables**												
** Gestational age groups**												
**FT**	**694,409**	**126,020 (18.2)**	**Ref.**	**Ref.**	**367,518**	**79,115 (21.5)**	**Ref.**	**Ref.**	**326,891**	**46,905 (14.4)**	**Ref.**	**Ref.**
**PT**	**38,348**	**9,980 (26.0)**	**1.62 (1.58–1.65)**	**1.23 (1.07–1.41)**	**20,944**	**6,153 (29.4)**	**1.55 (1.51–1.59)**	**1.08 (0.90–1.30)**	**17,404**	**3,827 (22.0)**	**1.72 (1.66–1.78)**	**1.51 (1.21–1.89)**
**MLPT**	**28,195**	**6,737 (23.9)**	**1.42 (1.39–1.46)**	**1.21 (1.04–1.41)**	**15,446**	**4,245 (27.5)**	**1.38 (1.34–1.43)**	**1.05 (0.86–1.28)**	**12,749**	**2,492 (19.6)**	**1.46 (1.41–1.52)**	**1.52 (1.20–1.93)**
**VPT**	**6,460**	**2,243 (34.7)**	**2.62 (2.52–2.74)**	**1.45 (1.03–2.05)**	**3,480**	**1,287 (37.0)**	**2.37 (2.24–2.50)**	**1.42 (0.93–2.16)**	**2,980**	**956 (32.1)**	**3.06 (2.87–3.26)**	**1.51 (0.82–2.79)**
** Birth year**												
**2011**	**246,499**	**35,652 (14.5)**	**Ref.**	**Ref.**	**130,852**	**22,192 (17.0)**	**Ref.**	**Ref.**	**115,647**	**13,460 (11.6)**	**Ref.**	**Ref.**
**2012**	**254,547**	**47,534 (18.7)**	**1.18 (1.17–1.20)**	**1.25 (1.16–0.34)**	**134,233**	**29,551 (22.0)**	**1.21 (1.19–1.23)**	**1.28 (1.17–1.40)**	**120,314**	**17,983 (15.0)**	**1.16 (1.13–1.19)**	**1.19 (1.06–1.34))**
**2013**	**231,711**	**52,814 (22.8)**	**1.96 (1.94–1.99)**	**2.20 (2.05–2.36)**	**123,377**	**33,525 (27.2)**	**2.00 (1.97–2.04)**	**2.13 (1.95–2.33)**	**108,334**	**19,289 (17.8)**	**1.92 (1.88–1.96)**	**2.34 (2.08–2.63)**
** Sex**												
**Boy**	**388,462**	**85,268 (22.0)**	**Ref.**	**Ref.**	**-**	**-**	**-**	**-**	**-**	**-**	**-**	**-**
**Girl**	**344,295**	**50,732 (14.7)**	**0.64 (0.64–0.65)**	**0.62 (0.59–0.65)**	**-**	**-**	**-**	**-**	**-**	**-**	**-**	**-**
** Cesarean section delivery**												
**No**	**456,660**	**82,230 (18.0)**	**Ref.**	**Ref.**	**241,041**	**51,631 (21.4)**	**Ref.**	**Ref.**	**215,619**	**30,599 (14.2)**	**Ref.**	**Ref.**
**Yes**	**276,097**	**53,770 (19.5)**	**1.10 (1.09–1.11)**	**1.02 (0.96–1.07)**	**147,421**	**33,637 (22.8)**	**1.08 (1.07–1.10)**	**1.02 (0.96–1.10)**	**128,676**	**20,133 (15.6)**	**1.12 (1.10–1.14)**	**0.99 (0.91–1.09)**
** Birth weight (kg)** ^**c**^ ^**)**^												
**Normal (≥2.5, <4.0)**	**649,485**	**116,649 (18.0)**	**Ref.**	**Ref.**	**344,263**	**73,514 (21.4)**	**Ref.**	**Ref.**	**305,222**	**43,135 (14.1)**	**Ref.**	**Ref.**
**Low (<2.5)**	**61,451**	**15,259 (24.8)**	**1.45 (1.42–1.47)**	**1.22 (1.10–1.36)**	**29,574**	**8,641 (29.2)**	**1.45 (1.42–1.48)**	**1.24 (1.08–1.42)**	**31,877**	**6,618 (20.8)**	**1.52 (1.48–1.56)**	**1.20 (1.02–1.41)**
**High (≥4.0)**	**21,765**	**4,083 (18.8)**	**1.07 (1.04–1.11)**	**0.89 (0.76–1.04)**	**14,597**	**3,107 (21.3)**	**1.02 (0.98–1.05)**	**0.85 (0.71–1.03)**	**7,168**	**976 (13.6)**	**0.99 (0.93–1.06)**	**0.97 (0.72–1.32)**
** Abnormality of growth** ^**d**^ ^**)**^												
**Normal**	**582,407**	**113,601 (19.5)**	**Ref.**	**Ref.**	**308,474**	**71,567 (23.2)**	**Ref.**	**Ref.**	**273,933**	**42,034 (15.3)**	**Ref.**	**Ref.**
**Abnormal**	**3,132**	**751 (24.0)**	**1.33 (1.24–1.43)**	**1.38 (0.98–1.95)**	**1,548**	**441 (28.5)**	**1.35 (1.23–1.48)**	**1.63 (1.06–2.50)**	**1,584**	**310 (19.6)**	**1.36 (1.22–1.53)**	**1.10 (0.63–1.95)**
** History of breastfeeding**												
**No**	**148,274**	**31,389 (21.2)**	**Ref.**	**Ref.**	**81,233**	**20,234 (24.9)**	**Ref.**	**Ref.**	**67,041**	**11,155 (16.6)**	**Ref.**	**Ref.**
**6month**	**111,358**	**20,102 (18.1)**	**0.83 (0.82–0.84)**	**0.88 (0.83–0.93)**	**59,594**	**12,746 (21.4)**	**0.83 (0.81–0.85)**	**0.90 (0.83–0.97)**	**51,764**	**7,356 (14.2)**	**0.84 (0.81–0.86)**	**0.84 (0.76–0.93)**
**1year**	**132,706**	**23,511 (17.7)**	**0.79 (0.78–0.81)**	**0.87 (0.81–0.92)**	**67,893**	**14,473 (21.3)**	**0.81 (0.79–0.83)**	**0.88 (0.81–0.96)**	**64,813**	**9,038 (13.9)**	**0.80 (0.78–0.82)**	**0.83 (0.75–0.93)**
**Maternal variables**												
** Age (years)**												
**<40**	**710,878**	**131,868 (18.6)**	**Ref.**	**Ref.**	**376,756**	**82,707 (22.0)**	**Ref.**	**Ref.**	**334,122**	**49,161 (14.7)**	**Ref.**	**Ref.**
**≥40**	**21,879**	**4,132 (18.9)**	**1.09 (1.06–1.12)**	**1.04 (0.94–1.15)**	**11,706**	**2,561 (21.9)**	**1.07 (1.03–1.11)**	**1.03 (0.91–1.17)**	**10,173**	**1,571 (15.4)**	**1.12 (1.07–1.18)**	**1.07 (0.91–1.27)**
** Socioeconomic status** ^**e**^ ^**)**^												
**1**	**95,424**	**19,558 (20.5)**	**Ref.**	**Ref.**	**50,706**	**12,227 (24.1)**	**Ref.**	**Ref.**	**44,718**	**7,331 (16.4)**	**Ref.**	**Ref.**
**2**	**125,079**	**24,853 (19.9)**	**0.95 (0.93–0.97)**	**0.86 (0.78–0.95)**	**66,328**	**15,474 (23.3)**	**0.95 (0.92–0.97)**	**0.89 (0.79–1.01)**	**58,751**	**9,379 (16.0)**	**0.95 (0.93–0.98)**	**0.82 (0.70–0.95)**
**3**	**192,121**	**36,486 (19.0)**	**0.91 (0.89–0.92)**	**0.85 (0.78–0.93)**	**101,579**	**22,785 (22.4)**	**0.91 (0.89–0.93)**	**0.86 (0.77–0.97)**	**90,542**	**13,701 (15.1)**	**0.91 (0.88–0.93)**	**0.83 (0.72–0.95)**
**4**	**205,872**	**36,365 (17.7)**	**0.85 (0.84–0.87)**	**0.82 (0.75–0.90)**	**109,166**	**22,897 (21.0)**	**0.86 (0.84–0.88)**	**0.85 (0.76–0.95)**	**96,706**	**13,468 (13.9)**	**0.85 (0.82–0.87)**	**0.79 (0.68–0.94)**
**5**	**110,043**	**18,041 (16.4)**	**0.83 (0.81–0.85)**	**0.85 (0.76–0.95)**	**58,422**	**11,442 (19.6)**	**0.84 (0.82–0.86)**	**0.89 (0.77–1.02)**	**51,621**	**6,599 (12.8)**	**0.81 (0.79–0.84)**	**0.80 (0.67–0.96)**
** Multiple gestation**												
**No**	**731,071**	**135,583 (18.6)**	**Ref.**	**Ref.**	**387,575**	**85,011 (21.9)**	**Ref.**	**Ref.**	**343,496**	**50,572 (14.7)**	**Ref.**	**Ref.**
**Yes**	**1,686**	**417 (24.7)**	**1.40 (1.27–1.54)**	**0.99 (0.85–1.16)**	**887**	**257 (29.0)**	**1.42 (1.26–1.61)**	**1.05 (0.87–1.27)**	**799**	**160 (20.0)**	**1.40 (1.19–1.63)**	**0.91 (0.71–1.17)**
** Preterm labor**												
**No**	**664,593**	**121,190 (18.2)**	**Ref.**	**Ref.**	**352,071**	**76,086 (21.6)**	**Ref.**	**Ref.**	**312,522**	**45,104 (14.4)**	**Ref.**	**Ref.**
**Yes**	**68,164**	**14,810 (21.7)**	**1.26 (1.24–1.28)**	**1.01 (0.92–1.10)**	**36,391**	**9,182 (25.2)**	**1.24 (1.22–1.27)**	**1.01 (0.91–1.13)**	**31,773**	**5,628 (17.7)**	**1.29 (1.26–1.33)**	**0.99 (0.85–1.16)**
** PROM**												
**No**	**710,791**	**130,749 (18.4)**	**Ref.**	**Ref.**	**376,482**	**82,028 (21.8)**	**Ref.**	**Ref.**	**334,309**	**48,721 (14.6)**	**Ref.**	**Ref.**
**Yes**	**21,966**	**5,251 (23.9)**	**1.39 (1.35–1.43)**	**1.28 (1.11–1.49)**	**11,980**	**3,240 (27.0)**	**1.33 (1.28–1.38)**	**1.31 (1.09–1.58)**	**9,986**	**2,011 (20.1)**	**1.47 (1.41–1.54)**	**1.25 (0.98–1.59)**
** Gestational diabetes**												
**No**	**654,607**	**120,639 (18.4)**	**Ref.**	**Ref.**	**346,574**	**75,534 (21.8)**	**Ref.**	**Ref.**	**308,033**	**45,105 (14.6)**	**Ref.**	**Ref.**
**Yes**	**78,150**	**15,361 (19.7)**	**1.07 (1.05–1.09)**	**1.03 (0.95–1.12)**	**41,918**	**9,764 (23.3)**	**1.07 (1.05–1.09)**	**1.04 (0.94–1.15)**	**36,262**	**5,627 (15.5)**	**1.06 (1.03–1.09)**	**1.02 (0.89–1.16)**
** Gestational hypertension**												
**No**	**723,103**	**133,842 (18.5)**	**Ref.**	**Ref.**	**383,552**	**83,963 (21.9)**	**Ref.**	**Ref.**	**339,551**	**49,879 (14.7)**	**Ref.**	**Ref.**
**Yes**	**9,654**	**2,158 (22.4)**	**1.23 (1.17–1.28)**	**1.14 (0.93–1.40)**	**4,910**	**1,305 (26.6)**	**1.23 (1.17–1.30)**	**1.10 (0.85–1.44)**	**4,744**	**853 (18.0)**	**1.24 (1.16–1.33)**	**1.18 (0.85–1.64)**
** Smoking during pregnancy**												
**No**	**60,703**	**10,663 (17.6)**	**Ref.**	**Ref.**	**32,029**	**6,681 (20.9)**	**Ref.**	**Ref.**	**28,584**	**3,892 (13.6)**	**Ref.**	**Ref.**
**Yes**	**515**	**134 (26.0)**	**1.60 (1.35–1.90)**	**1.49 (1.17–1.89)**	**288**	**85 (29.5)**	**1.57 (1.27–1.95)**	**1.47 (1.08–1.99)**	**227**	**49 (21.6)**	**1.63 (1.23–2.15)**	**1.48 (0.99–2.20)**

Abbreviations: NI, Neurodevelopmental impairments; FT, Full term; PT, Preterm; MLPT, Moderate-to-late preterm; VPT, Very-preterm; PROM, Premature rupture of membranes.

^a^Crude.

^b^Adjusted for gestational age groups, birth year, sex, cesarean section delivery, birth weight, abnormality of growth, history of breastfeeding, maternal age, socioeconomic status, multiple gestation, preterm labor, PROM, gestational diabetes, gestational hypertension, and smoking during pregnancy.

^c^Classified by the criteria for normal, low, and high birth weight according to the WHO recommendation [20].

^d^Classified as normal (having both normal weight and height) and abnormal (either abnormal weight or abnormal height) based on the weight and height data measured at 4th screening following the growth curve suggested by Korean National Growth Charts for Children and Adolescents [21].

^e^National Health Insurance Premium quintiles (highest to lowest from 5 to 1).

The risk of NI increased with increasing birth year from 2011 to 2013 and was doubled in 2013 compared to 2011 (all: HR, 2.20 [95% CI, 2.05 to 2.36]; boys: HR, 2.13 [95% CI, 1.95 to 2.33]; girls: HR, 2.34 [95% CI, 2.08 to 2.63]). The risk was also increased in children with low birth weight (all: HR, 1.22 [95% CI, 1.10 to 1.36]; boys: HR, 1.24 [95% CI, 1.08 to 1.42]; girls: HR, 1.20 [95% CI, 1.02 to 1.41]), PROM (all: HR, 1.28 [95% CI, 1.11 to 1.49]; boys: HR, 1.31 [95% CI, 1.09 to 1.58], and maternal smoking during pregnancy (all: HR, 1.49 [95% CI, 1.17 to 1.89]; boys: HR, 1.47 [95% CI, 1.08 to 1.99]).

However, both a longer duration of breastfeeding (all: HR, 0.87 [95% CI, 0.81 to 0.92]; boys: HR, 0.88 [95% CI, 0.81 to 0.96]; girls: HR, 0.83 [95% CI, 0.75 to 0.93]) and higher socioeconomic status (all: HR, 0.85 [95% CI, 0.76 to 0.95]; boys: HR, 0.85 [95% CI, 0.76 to 0.95]; girls: HR, 0.80 [95% CI, 0.67 to 0.96]) were associated with reduced risk (Table 2).

Although the HRs of children born MLPT and VPT were elevated in a similar manner to the risk of overall NI, children born VPT showed statistically significant elevations in the incidence of NI for gross motor skills (HR, 2.15; 95% CI, 1.38 to 3.34) and fine motor skills (HR, 1.89; 95% CI, 1.18 to 3.04). Also, children born MLPT showed statistically significant elevations in the incidence of NI for only gross motor skills (HR, 1.44; 95% CI, 1.16 to 1.80Obstetric factors, such as PROM and gestational hypertension, were independent risk factors for impaired problem-solving skills and self-help skills of children (S1 and S2 Tables).

## Discussion

Based on the current study, the risk of NI was higher in children born MLPT, as well as in children born VPT, than in those born FT. This study had a large-scale population-based retrospective cohort design based on data linkage between the NHSPIC and NHISS healthcare claims data in Korea. The risk was correspondingly shown to be increased in the motor skills (gross and fine) and social-cognitive (problem-solving, communication, and self-help) domains of NI, although only the motor skills did reach statistical significance. These are crucial and necessary findings due to the lack of studies on the impact of MLPT births on NI in young children, as well as the continuously increasing frequency of MLPT births. Furthermore, to the authors’ knowledge, this is the first study in Korea to investigate the impact of both MLPT and VPT births on NI using a large-scale population-based cohort design. Although a few similar studies have been performed in other countries, most have limitations including study design, short-term follow-up period, small sample size, and lack of comprehensiveness regarding NI domains to be considered to confirm the impact of MLPT birth on NI. Therefore, the results of the present study form a potential basis for the development of intervention strategies for PT births and related impairments of young children in other countries, as well as in Korea.

Although the risk of NI is well known to increase along with a decrease in gestational age, and most of the risk of NI has been identified in children born VPT, the current study confirmed that the risk of NI is still quite elevated in children born MLPT relative to those born FT (HR, 1.21; 95% CI, 1.04 to 1.41). A large population-based cohort study in China of preschoolers aged 3–5 years suggested a non-linear association of gestational age with neurodevelopmental delay. The risk of neurodevelopmental delay was elevated before 40 gestational weeks but lowered afterwards, indicating that neurodevelopmental outcomes benefit from longer gestational age [4]. Those study results underscore impacts on the risk of suspected developmental delay regarding communication, problem-solving, personal social behaviors, gross motor skills, and fine motor skills corresponding to the current results, although the current study had a longer follow-up period of 6 years after birth. Another population-based cohort study was conducted in rural China for children of 1–59 months, one in the United Kingdom for children of 2 years, and another in Chile for children of 8 and 18 months, all of which had relatively shorter follow-up periods than the current study, showed similar results to the current one [10, 16, 23]. In particular, the study in the East Midlands of the United Kingdom found that children born MLPT had 2.19 times higher risk of neurodevelopmental disability than children born FT (95% CI, 1.27 to 3.75) [23]. Therefore, regarding the adverse effect of MLPT births on NI identified in the current study and alignment with previous studies, careful and close monitoring of NI should be emphasized for young children born MLPT as well as VPT.

In the current study, to observe the independent effect of gestational age on NI in young children, covariates such as birth year, sex, cesarean section delivery, birth weight, duration of breastfeeding, maternal age, socioeconomic status, and multiple gestation, preterm labor, PROM, gestational diabetes, gestational hypertension, and smoking during pregnancy were included in the adjusted model to evaluate the impact of EPT and MLPT births on NI.

Low birth weight (<2.5 kg) has already been found to be associated with a high risk of NI along with premature birth in several previous studies [5, 24]. The present study confirmed that low birth weight was associated with high NI risk—both overall and in five specific NI domains—independently of other covariates, including preterm birth. Furthermore, inappropriate weight or height measurements in children between 30 and 36 months of age were associated with an increased risk of overall and specific domains of NI in those children as well. A recent meta-analysis suggested that the height-for-age z-score is positively associated with cognitive and motor development [25], which corresponds to the current study results. These results indicate that proper growth may be an important factor in promoting child neurodevelopment. In particular, the interval from birth to 36 months is known to be the growth catch-up period for high-risk newborns without completion of intrauterine growth before birth [26]. Therefore, weight or height measurements during this period could be highly meaningful data that reflect the child’s health status after birth, and growth abnormalities should be treated with sensitivity during this period.

PROM is the most common antecedent of preterm birth, and gestational age at birth following PROM is related to the child’s neurological prognosis [27]. However, in this study, PROM was identified as an independent risk factor for NI even after adjusting for gestational age and other covariates. This result supports a previous study [28], which showed that preterm infants born after PROM had a higher risk of severe NI at 2 years of age than infants born after spontaneous preterm labor with intact membranes. This result is also similar to the findings of a prior study [29] that prolonged PROM increased the risk of developmental problems measured using the ASQ at in children born MLPT aged 4 and 5. However, there is a need to elucidate the relationship between these two variables through further studies, such as prospective long-term studies, since there is still a weak theoretical basis for a causal relationship between PROM and children’s neurological prognosis. Furthermore, in the current study, maternal gestational hypertension increased the risk for NI in the social-cognitive (problem-solving and self-help) domain. This echoes the finding that maternal hypertension was associated with a long-term decrease in verbal ability in children aged 10 years on average even after adjusting for birth weight and gestational age [30]. An explanation for this finding is that physiological factors interfere with prenatal brain development by reducing oxygen supply and nutrient supply through placental blood flow in hypertensive diseases of pregnancy [31], and caution is required regarding about long-term neurodevelopment in children born to mothers with gestational hypertension.

One interesting finding from the current study is that children who breastfed until either 6 months or 12 months after birth had a lower risk of NI than children without breastfeeding experience after adjustment for all other covariates. During infancy, breastfeeding is emphasized as a major strategy to promote growth and development even in healthy young children [27]. Therefore, the WHO and United Nations Children’s Fund recommend exclusive breastfeeding for up to 6 months [32] given the evidence that exclusive breastfeeding during the first 3–6 months of life is strongly associated with higher ASQ version 3 scores, especially in the domains of communication, gross motor skills, and problem-solving [33]. Another study conducted to assess the effect of breastfeeding for longer than 6 months indicated that it increased Bayley scale scores in cognitive, language, and motor development [34]. In the current study results, although children breastfed for more than 12 months had a slightly lower risk of neurodevelopmental delay than children breastfed for 6 months, no meaningful risk difference was observed between them overall and in all domains of NI. Regarding both these and previous study results, breastfeeding for at least 6 months after birth should be considered an intervention to support appropriate growth and development in early life. The rate of breastfeeding in Korea has been declining since 2012, and the rate of exclusive breastfeeding until 6 months of age is 18.3%, which is very low compared to the international average of 38% [35]. Furthermore, in Korea, postpartum care centers, which are used by many Korean mothers to recover after childbirth, are often environments in which mothers and children are separated, so the breastfeeding rate is particularly low for the first 1 to 2 weeks after birth [36]. Therefore, it is necessary to identify the obstacles to breastfeeding and to develop thorough strategies to improve the rate of breastfeeding according to the specific situation in Korea.

As is well-known from recent study results, the socioeconomic status of parents, particularly mothers, was the most important factor in areas of child neurodevelopment such as academic performance, intelligence quotient, and growth rate. The current study showed similar results. Therefore, low socioeconomic status deleteriously affects children’s neurodevelopment [37, 38]. In addition, parental socioeconomic status can play an important role in whether children are provided appropriate sociocultural and economic environments, including accessibility and support for medical services, education, food, and other daily expenses, which could impact growth and development both before and after birth.

Male sex and recent birth year were also independent factors associated with increased NI risk in young children, which was consistent across all five domains in the current study. Male sex had been previously identified as a risk factor for NI [39, 40], which has been explained by the vulnerability of boys to the risk of developmental impairments in early childhood [41]. The increased risk from 2011 to 2013 may be associated with the increase in newborns with risk factors, such as premature birth and multiple gestation, due to the recent increase in artificial insemination in response to infertility. However, further studies of this issue are required.

In this study, maternal smoking during pregnancy was identified as an independent factor that significantly increased the risk of NI despite the very small number of cases. This result supports the findings of previous studies, according to which maternal smoking during pregnancy had a long-term negative effect on children’s cognitive development [42, 43] and neurobehavioral abnormalities [44] later in life. These findings can be interpreted as suggesting that exposure to cigarettes during fetal life leads to defects in the development of the child’s central nervous system. Therefore, maternal smoking during pregnancy should be emphasized as a modifiable risk factor associated with cognitive problems in children.

## Conclusion and practical implications

This study confirmed that children born MLPT and VPT had higher risks of NI than those born FT, based on nationally representative large-scale population data from NHSPIC and NHISS. In addition, a later birth year, male sex, low birth weight, and abnormal growth, which were investigated as covariates, were child-related factors that increased the risk of NI, and a longer duration of breastfeeding and higher socioeconomic status were found to lower the risk of NI. A high-risk obstetric history (i.e., PROM) and smoking during pregnancy, as a maternal health behavior, were also associated with an increased risk of NI.

Based on the results of this study, we can make the following suggestions. Healthcare professionals must take into account the child’s gestational age and birth weight when caring for young children and providing parental counseling and education regarding the risk of NI. In particular, the low clinical and social interest in children born with MLPT hinders the provision of appropriate care for children born with MLPT and their families [45], so healthcare professionals should pay special attention to these children and manage them in a way that promotes their optimal growth and development. In addition, socioeconomic status must be investigated to support the neurodevelopmental development of young children, and social and national systematic support for the optimal development of low-income children is strongly emphasized. In particular, high socioeconomic status has been found to be strongly associated with breastfeeding practice in many countries [46–49], and in this study, the duration of breastfeeding and socioeconomic level were important independent factors affecting the risk of NI. A possible explanation for these results is that wealthier mothers may practice breastfeeding for a longer period of time, thereby contributing to their children’s better neurodevelopment outcomes. Therefore, policy and practical strategies are required for short-term success and long-term implementation of breastfeeding for mothers and families of low socioeconomic status. In this study, PROM was found to have an independent relationship with long-term NI, and PROM is a very common cause of preterm delivery, accounting for approximately 30% of all preterm births [50]. Although the cause of PROM has not yet been clearly identified, potential risk factors include smoking, sexual activity, dietary or nutritional deficiencies, vaginal bleeding, multiple gestation, a history of PROM and preterm delivery in a previous pregnancy, and genital tract infection [51]. Therefore, close and careful management by health professionals to prevent PROM is necessary for mothers at high risk of PROM, and education to prevent spontaneous premature birth due to PROM needs to be provided in pregnancy and childbirth education. Additionally, close follow-up and management by health professionals is required for NI in children born with PROM. In addition, this study emphasized the importance of healthcare professionals’ management of maternal health behaviors, such as maternal smoking, that can affect the neural development of the fetus during pregnancy.

## Limitations and suggestions for future research

The current study presents the impacts of VPT and MLPT births on NIs in young children with a large population-based long-term follow-up cohort study design. Some limitations are as follows. First, 0.8% of the newborns enrolled from 2011 to 2013 were excluded from the final analysis, as they were missing information from the second to seventh developmental screening tests. Additionally, since not all children participated in all rounds of the NHSPIC, it was not possible to generate follow-up results on neurodevelopmental changes based on repeated evaluation in each round of the NHSPIC. In addition, because preterm birth is the result of a very complex and unpredictable mechanism, our results may have inherent limitations in fully explaining the causal relationship regarding the impact of MLPT and VPT births on NI. Additionally, in this study, NI was derived based on the results of screening tests of K-ASQ and K-DST rather than a diagnostic test; thus, it is possible that children identified as having NI did not actually have a developmental delay or disability. Therefore, caution must be taken when interpreting the results of this study. Since the K-ASQ and K-DST are evaluated based on parental reports, there may be limitations in ensuring the objectivity of test results due to parents’ subjective and lenient evaluations. However, this approach was chosen to improve the reliability of the test results since these assessments involve a process of reassessing and finalizing the child’s development level by a doctor at the medical institution responsible for the examination. Finally, the data in this study were obtained through a retrospective approach, meaning that there were limitations in securing some data that could have a causal relationship with children’s neurodevelopment. In particular, certain potential factors (including maternal health behaviors such as physical activity, drinking, and gestational weight gain) that could be associated with both PT and NI were not considered because of data incompleteness. Therefore, follow-up research should conduct a prospective longitudinal investigation to establish causal relationships between PT and NI.

## Supporting information

S1 Table The HRs and 95%CI on neurodevelopmental impairments in motor skills. (DOCX)

S2 Table The HRs and 95%CI on neurodevelopmental impairments in social-cognitive skills. (DOCX)

## References

[1] Walani SR. Global burden of preterm birth. BJOG: Int Journal Obs & Gynae. 2020;150(1): 31–33. doi: 10.1002/ijgo.13195 32524596

[2] Allotey J, Zamora J, Cheong‐See F, Kalidindi M, Arroyo‐Manzano D, Asztalos E, et al. Cognitive, motor, behavioural and academic performances of children born preterm: a meta‐analysis and systematic review involving 64 061 children. BJOG: Int Journal Obs & Gynae. 2018;125(1): 16–25. doi: 10.1111/1471-0528.14832 29024294

[3] World Health Organization. Preterm birth [Internet]. 2022 Nov 14 [cited 25 Jan 2023]. Available from: https://www.who.int/news-room/fact-sheets/detail/preterm-birth.

[4] Hua J, Barnett AL, Lin Y, Guan H, Sun Y, Williams GJ, et al. Association of gestational age at birth with subsequent neurodevelopment in early childhood: a national retrospective cohort study in China. Front Pediatr. 2022;10: 860192. doi: 10.3389/fped.2022.860192 35712637 PMC9194570

[5] Duncan AF, Matthews MA. Neurodevelopmental outcomes in early childhood. Clin Perinatol. 2018;45(3): 377–392. doi: 10.1016/j.clp.2018.05.001 30144844

[6] Rogers EE, Hintz SR. Early neurodevelopmental outcomes of extremely preterm infants. Semin Perinatol. 2016; 40(8): 497–509. doi: 10.1053/j.semperi.2016.09.002 27865437

[7] Walsh JM, Doyle LW, Anderson PJ, Lee KJ, Cheong JL. Moderate and late preterm birth: effect on brain size and maturation at term-equivalent age. Radiology. 2014;273(1): 232–240. doi: 10.1148/radiol.14132410 24914576

[8] Boswinkel V, Nijboer-Oosterveld J, Nijholt IM, Edens MA, Mulder-de Tollenaer SM, Boomsma MF, et al. A systematic review on brain injury and altered brain development in moderate-late preterm infants. Early Hum Dev. 2020;148: 105094. doi: 10.1016/j.earlhumdev.2020.105094 32711341

[9] Hirvonen M, Ojala R, Korhonen P, Haataja P, Eriksson K, Gissler M, et al. Cerebral palsy among children born moderately and late preterm. Pediatrics 2014;134(6): e1584–e1593. doi: 10.1542/peds.2014-0945 25422011

[10] Schonhaut L, Armijo I, Pérez M. Gestational age and developmental risk in moderately and late preterm and early term infants. Pediatrics. 2015;135(4): e835–e841. doi: 10.1542/peds.2014-1957 25733752

[11] de Jong M, Verhoeven M, Lasham CA, Meijssen CB, van Baar AL. Behaviour and development in 24-month-old moderately preterm toddlers. Arch Dis Child. 2015;100(6): 548–553. doi: 10.1136/archdischild-2014-307016 25589560

[12] Cheong JL, Thompson DK, Spittle AJ, Potter CR, Walsh JM, Burnett AC, et al. Brain volumes at term-equivalent age are associated with 2-year neurodevelopment in moderate and late preterm children. J Pediatr. 2016;174: 91–97. e1. doi: 10.1016/j.jpeds.2016.04.002 27174146

[13] Cheong J, Cameron KLI, Thompson D, Anderson PJ, Ranganathan S, Clark R, et al. Impact of moderate and late preterm birth on neurodevelopment, brain development and respiratory health at school age: protocol for a longitudinal cohort study (LaPrem study). BMJ open. 2021;11(1): e044491. doi: 10.1136/bmjopen-2020-044491 33518527 PMC7852967

[14] Woythaler M, McCormick MC, Mao W-Y, Smith VC. Late preterm infants and neurodevelopmental outcomes at kindergarten. Pediatrics. 2015;136(3): 424–431. doi: 10.1542/peds.2014-4043 26260723

[15] Jin JH, Yoon SW, Song J, Kim SW, Chung HJ. Long-term cognitive, executive, and behavioral outcomes of moderate and late preterm at school age. Clin Exp Pediatr. 2020;63(6): 219. doi: 10.3345/kjp.2019.00647 32024339 PMC7303421

[16] Zhou H, Qu X, Yang Y, Kc A, Liu X, Yang C, et al. Relationship between moderate to late preterm, diet types and developmental delay in less-developed rural China. Nutr Neurosci. 2022;25(1): 70–79. doi: 10.1080/1028415X.2020.1712534 31973664

[17] Korean Statistical Information Service. Live birth by city, province/duration of pregnancy [Internet]. 2022 Aug 24 [cited 25 Jan 2023]. https://kosis.kr/statHtml/statHtml.do?orgId=101&tblId=DT_1B81A15&conn_path=I2.

[18] Korea Disease Control and Prevention Agency. 2021 Manual for Consulting Doctors for Health Screening Program for Infants and Children. [Internet]. 2021 Dec 14 [cited 9 Oct 2023]. https://health.kdca.go.kr/healthinfo/biz/health/ntcnInfo/helthEdcRecsroom/helthEdcRecsroomView.do?phledu_recsroom_sn=1736.

[19] Korean Statistical Information on Service. Status of infant health screening targets and number of examinees by job title and gender. [Internet]. 2023 Oct 9 [cited 9 Oct 2023]. https://kosis.kr/statHtml/statHtml.do?orgId=350&tblId=DT_35007_N147&conn_path=I2

[20] World Health Organization. ICD-10: International Statistical Classification of Diseases and Related Health Problems: tenth revision, 2nd ed. World Health Organization; 2004.

[21] Kim JH, Yun S, Hwang SS, Shim JO, Chae HW, Lee YJ, et al. Committee for the Development of Growth Standards for Korean Children and Adolescents; Committee for School Health and Public Health Statistics, the Korean Pediatric Society; Division of Health and Nutrition Survey, Korea Centers for Disease Control and Prevention. The 2017 Korean National Growth Charts for children and adolescents: development, improvement, and prospects. Korean J Pediatr. 2018;61(5): 135–149. 29853938 10.3345/kjp.2018.61.5.135 PMC5976563

[22] Korea Centers for Disease Control and Prevention & The Korean Pediatric Society. Guidelines for the use of the Korean Developmental Screening Test for Infants & Children [Internet]. 2014 Oct 15 [cited 13 Feb 2023]. http://www.cdc.go.kr/board/board.es?mid=a20507020000&bid=0019&act=view&list_no=138232.

[23] Johnson S, Matthews R, Draper ES, Field DJ, Manktelow BN, Marlow N, et al. Early emergence of delayed social competence in infants born late and moderately preterm. J Dev Behav Pediatr. 2015;36(9): 690–699. doi: 10.1097/DBP.0000000000000222 26461097

[24] Oudgenoeg-Paz O, Mulder H, Jongmans MJ, van der Ham IJ, Van der Stigchel S. The link between motor and cognitive development in children born preterm and/or with low birth weight: A review of current evidence. Neurosci Biobehav Rev. 2017;80: 382–393. doi: 10.1016/j.neubiorev.2017.06.009 28642071

[25] Sudfeld CR, Charles McCoy D, Danaei G, Fink G, Ezzati M, Andrews KG, et al. Linear growth and child development in low-and middle-income countries: a meta-analysis. Pediatrics. 2015;135(5): e1266–e1275. doi: 10.1542/peds.2014-3111 25847806

[26] Vizzari G, Morniroli D, Tiraferri V, Macchi M, Gangi S, Consales A, et al. Postnatal growth of small for gestational age late preterm infants: determinants of catch-up growth. Pediatr Res. 2022;1–6. doi: 10.1038/s41390-022-02402-3 36460739 PMC10356607

[27] Lorthe E, Torchin H, Delorme P, Ancel PY, Marchand-Martin L, Foix-L’Hélias L, et al. Preterm premature rupture of membranes at 22–25 weeks’ gestation: perinatal and 2-year outcomes within a national population-based study (EPIPAGE-2). Am J Obstet Gynecol. 2018;219(3): 298.e1–298.e14. doi: 10.1016/j.ajog.2018.05.029 29852153

[28] Spinillo A, Capuzzo E, Stronati M, Ometto A, Orcesi S, Fazzi E. Effect of preterm premature rupture of membranes on neurodevelopmental outcome: follow up at two years of age. Br J Obstet Gynaecol. 1995;102(11): 882–887. doi: 10.1111/j.1471-0528.1995.tb10875.x 8534623

[29] Bos AF, Hornman J, de Winter AF, Reijneveld SA. Predictors of persistent and changing developmental problems of preterm children. Early Hum Dev. 2021;156: 105350. doi: 10.1016/j.earlhumdev.2021.105350 33780801

[30] Whitehouse AJ, Robinson M, Newnham JP, Pennell CE. Do hypertensive diseases of pregnancy disrupt neurocognitive development in offspring? Paediatr Perinat Epidemiol. 2012;26(2): 101–108. doi: 10.1111/j.1365-3016.2011.01257.x 22324495

[31] Rees S, Harding R, Walker D. An adverse intrauterine environment: implications for injury and altered development of the brain. Int J Dev Neurosci. 2008;26(1): 3–11. doi: 10.1016/j.ijdevneu.2007.08.020 17981423

[32] World Health Organization. Exclusive breastfeeding for six months best for babies everywhere [Internet]. 2011 Jan 15 [cited 25 Jan 2023]. https://www.who.int/news/item/15-01-2011-exclusive-breastfeeding-for-six-months-best-for-babies-everywhere.

[33] Onyango S, Kimani-Murage E, Kitsao-Wekulo P, Langat NK, Okelo K, Obong’o C, et al. Associations between exclusive breastfeeding duration and children’s developmental outcomes: Evidence from Siaya county, Kenya. PLoS One. 2022;17(3): e0265366. doi: 10.1371/journal.pone.0265366 35358207 PMC8970373

[34] Leventakou V, Roumeliotaki T, Koutra K, Vassilaki M, Mantzouranis E, Bitsios P, et al. Breastfeeding duration and cognitive, language and motor development at 18 months of age: Rhea mother–child cohort in Crete, Greece. J Epidemiol Community Health. 2015;69(3): 232–239. 24336236 10.1136/jech-2013-202500

[35] Choi EJ, Park EJ, Kim HR, Oh MA, Lee NH, Choi JH. A Survey on actual condition of breast-feeding in Korea. 2016. Korean Committee for UNICEF. Korea Institute for Health and Social Affairs. https://www.unicef.or.kr/data/upload/ebook/crc-publications/609/.

[36] The Academy of Breastfeeding Medicine Korea. Why has breastfeeding rates declined in the last 10 years? [Internet]. 2022 Jun 27 [cited 25 Jan 2023]. http://www.bfmed.co.kr/re/sub/mbi_read.html?cate=1&idx=948.

[37] Poulain T, Vogel M, Kiess W. Review on the role of socioeconomic status in child health and development. Curr Opin Pediatr. 2020;32(2): 308–314. doi: 10.1097/MOP.0000000000000876 31895161

[38] Baharvand P, Nejad EB, Karami K, Amraei M. A review study of the role of socioeconomic status and its components in children’s health. GJMPBU. 2021;16(9): 1–9.

[39] El Elella SSA, Tawfik MA, El Fotoh WMMA, Barseem NF. Screening for developmental delay in preschool-aged children using parent-completed Ages and Stages Questionnaires: additional insights into child development. Postgrad Med J. 2017;93(1104): 597–602. doi: 10.1136/postgradmedj-2016-134694 28408725

[40] Valla L, Birkeland M, Hofoss D, Slinning K. Developmental pathways in infants from 4 to 24 months. Child Care Health Dev. 2017;43(4): 546–555. 28386948 10.1111/cch.12467

[41] Curtin M, Madden J, Staines A, Perry IJ. Determinants of vulnerability in early childhood development in Ireland: a cross-sectional study. BMJ open. 2013;3(5): e002387. doi: 10.1136/bmjopen-2012-002387 23674442 PMC3657679

[42] Clifford A, Lang L, Chen R. Effects of maternal cigarette smoking during pregnancy on cognitive parameters of children and young adults: a literature review. Neurotoxicol Teratol. 2012;34(6): 560–570. doi: 10.1016/j.ntt.2012.09.004 23022448

[43] Corrêa ML, da Silva BGC, Wehrmeister FC, Horta BL, Gonçalves H, Anselmi L, et al. Maternal smoking during pregnancy and intelligence quotient of offspring aged 18 and 30 years: Evidence from two birth cohorts in southern Brazil. Prev Med. 2022;156: 106983. 35150754 10.1016/j.ypmed.2022.106983

[44] Froggatt S, Reissland N, Covey J. The effects of prenatal cigarette and e-cigarette exposure on infant neurobehaviour: A comparison to a control group. EClinicalMedicine. 2020;28: 100602. doi: 10.1016/j.eclinm.2020.100602 33294816 PMC7700948

[45] Lee S. Parenting experiences of mothers of moderate-to-late preterm children in South Korea: a qualitative study. Child Health Nurs Res. 2022 Oct;28(4): 247–258. doi: 10.4094/chnr.2022.28.4.247 36379601 PMC9672526

[46] Moran-Lev H, Farhi A, Bauer S, Nehama H, Yerushalmy-Feler A, Mandel D, et al. Association of Socioeconomic Factors and Infant Nutrition Decisions: Breastfeeding and Type of Formula. Breastfeed Med. 2021;16(7): 553–557. doi: 10.1089/bfm.2020.0398 33835839

[47] Sarki M, Parlesak A, Robertson A. Comparison of national cross-sectional breast-feeding surveys by maternal education in Europe (2006–2016). Public Health Nutr;22(5): 848–861. doi: 10.1017/S1368980018002999 30516455 PMC6474715

[48] Santika O, Februhartanty J, Ariawan I. Feeding practices of young children aged 12–23 months in different socio-economic settings: a study from an urban area of Indonesia. Br J Nutr. 2016;116 Suppl 1:S1–S7. doi: 10.1017/S0007114515003438 26388172

[49] Byun JH , Lee JS, Kim TH. Association between breastfeeding and parental socioeconomic status: Analysis of the Korean national health and nutrition examination survey, 2013–2017. J Korean Soc Matern Child Health. 2021;25(1): 21–30.

[50] Robinson JN, Norwitz ER. Spontaneous preterm birth: Overview of risk factors and prognosis. [Internet]. 2023 Jul 14 [cited 25 Jan 2023]. https://www.uptodate.com/contents/spontaneous-preterm-birth-overview-of-risk-factors-and-prognosis

[51] Lee T, Silver H. Etiology and epidemiology of preterm premature rupture of the membranes. Clin Perinatol. 2001;28(4): 721–734. doi: 10.1016/s0095-5108(03)00073-3 11817185

