# Supplementary material for: Impact of moderate-to-late preterm birth on neurodevelopmental outcomes in young children: Results from retrospective longitudinal follow-up with nationally representative data
Source: PLoS One. 2023 Nov 16;18(11):e0294435. doi: 10.1371/journal.pone.0294435 (PMC10653423; doi:10.1371/journal.pone.0294435)
Supplement: S1 Table — (DOCX) [file pone.0294435.s001.docx]

**S1 Table. The HRs and 95%CI on Neurodevelopmental Impairments in Motor Skills**

| **Variables** | **Gross motor skills** | | | | **Fine motor skills** | | | | |
| --- | --- | --- | --- | --- | --- | --- | --- | --- | --- |
|  | **Total** | **Impairments**  **(n, %)** | **HR (95%CI)^a)^** | **HR (95%CI)^b)^** | **Total** | **Impairments**  **(n, %)** | | **HR (95%CI)^a)^** | **HR (95%CI)^b)^** |
| **Children variables** |  | | | | | | | | |
| **Gestational age groups** |  | | | | | | | | |
| FT | 694,406 | 47,838 (6.9) | Ref. | Ref. | 694,404 | 49,325 (7.1) | | Ref. | Ref. |
| PT | 38,348 | 4,914 (12.8) | 2.06 (2.00-2.12) | 1.58 (1.30-1.93) | 38,348 | 4,390 (11.5) | | 1.80 (1.75-1.86) | 1.23 (0.99-1.53) |
| MLPT | 28,195 | 3,000 (10.6) | 1.65 (1.59-1.71) | 1.44 (1.16-1.80) | 28,195 | 2,822 (10.0) | | 1.51 (1.46-1.57) | 1.20 (0.94-1.52) |
| VPT | 6,460 | 1,405 (21.8) | 4.12 (3.91-4.35) | 2.15 (1.38-3.34) | 6,460 | 1,122 (17.4) | | 3.28 (3.09-3.48) | 1.89 (1.18-3.04) |
| **Birth year** |  | | | | | | | | |
| 2011 | 246,496 | 14,831 (6.0) | Ref. | Ref. | 246,494 | 14,621 (5.9) | | Ref. | Ref. |
| 2012 | 254,547 | 18,726 (7.4) | 1.14 (1.12-1.17) | 1.25 (1.12-1.39) | 254,547 | 18,747 (7.4) | | 1.16 (1.13-1.18) | 1.27 (1.13-1.42) |
| 2013 | 231,711 | 19,195 (8.3) | 1.63 (1.60-1.67) | 1.83 (1.64-2.05) | 231,711 | 20,347 (8.8) | | 1.77 (1.73-1.81) | 2.17 (1.94-2.43) |
| **Sex** |  | | | | | | | | |
| Boy | 388,459 | 28,238 (7.3) | Ref. | Ref. | 388,459 | 37,805 (9.7) | | Ref. | Ref. |
| Girl | 344,295 | 24,514 (7.1) | 0.94 (0.93-0.96) | 0.89 (0.82-0.96) | 344,293 | 15,910 (4.6) | | 0.46 (0.45-0.46) | 0.40 (0.37-0.44) |
| **Birth weight (kg)^c)^** |  | | | | | | | | |
| Normal (2.5≤, <4.0) | 649,483 | 44,144 (6.8) | Ref. | Ref. | 649,480 | 45,509 (7.0) | | Ref. | Ref. |
| Low (<2.5) | 61,450 | 7,182 (11.7) | 1.79 (1.74-1.83) | 1.26 (1.08-1.47) | 61,451 | 6,588 (10.7) | | 1.60 (1.56-1.64) | 1.30 (1.11-1.53) |
| High (≥4.0) | 21,765 | 1,421 (6.5) | 0.98 (0.93-1.04) | 0.87 (0.68-1.12) | 21,765 | 1,612 (7.4) | | 1.08 (1.03-1.14) | 0.78 (0.61-1.01) |
| **Cesarean section delivery** |  |  |  |  |  |  | |  |  |
| No | 456,658 | 31,387 (6.9) | Ref. | Ref. | 456,658 | 32,310 (7.1) | | Ref. | Ref. |
| Yes | 276,096 | 21,365 (7.7) | 1.14 (1.12-1.16) | 1.07 (0.98-1.16) | 276,094 | 21,405 (7.8) | | 1.11 (1.09-1.13) | 1.08 (0.99-1.18) |
| **Abnormality of growth^d)^** |  | | | | | | | | |
| Normal | 582,407 | 43,350 (7.4) | Ref. | Ref. | 582,407 | 44,914 (7.7) | | Ref. | Ref. |
| Abnormal | 3,132 | 313 (10.0) | 1.44 (1.29-1.61) | 2.00 (1.29-3.11) | 3,132 | 290 (9.3) | | 1.28 (1.14-1.44) | 1.81 (1.11-2.96) |
| **History of breastfeeding** |  | | | | | | | | |
| No | 148,274 | 12,472 (8.4) | Ref. | Ref. | 148,274 | 12,269 (8.3) | | Ref. | Ref. |
| 6month | 111,357 | 7,713 (6.9) | 0.80 (0.78-0.83) | 0.90 (0.82-0.99) | 111,357 | 7,685 (6.9) | | 0.81 (0.79-0.84) | 0.94 (0.85-1.03) |
| 1year | 132,706 | 9,405 (7.1) | 0.81 (0.78-0.83) | 0.87 (0.79-0.97) | 132,706 | 8,635 (6.5) | | 0.75 (0.73-0.77) | 0.90 (0.81-1.00) |
| **Maternal variables** |  | | | | | | | | |
| **Age (years)** |  | | | | | | | | |
| <40 | 710,875 | 51,021 (7.2) | Ref. | Ref. | 710,873 | 52,062 (7.3) | | Ref. | Ref. |
| ≥40 | 21,879 | 1,731 (7.9) | 1.17 (1.11-1.23) | 0.96 (0.81-1.13) | 21,879 | 1,653 (7.6) | | 1.10 (1.05-1.15) | 1.04 (0.88-1.22) |
| **Socioeconomic status^e)^** |  | | | | | | | | |
| 1 | 95,424 | 7,565 (7.9) | Ref. | Ref. | 95,424 | 7,983 (8.4) | | Ref. | Ref. |
| 2 | 125,077 | 9,643 (7.7) | 0.96 (0.93-0.98) | 0.84 (0.72-0.98) | 125,079 | 10,186 (8.1) | | 0.96 (0.93-0.98) | 0.87 (0.74-1.01) |
| 3 | 192,120 | 14,047 (7.3) | 0.91 (0.88-0.93) | 0.88 (0.77-1.01) | 192,118 | 14,465 (7.5) | | 0.88 (0.86-0.91) | 0.90 (0.78-1.03) |
| 4 | 205,872 | 14,224 (6.9) | 0.86 (0.84-0.89) | 0.87 (0.76-1.00) | 205,870 | 13,955 (6.8) | | 0.80 (0.78-0.82) | 0.79 (0.68-0.91) |
| 5 | 110,043 | 7,028 (6.4) | 0.83 (0.81-0.86) | 0.81 (0.68-0.97) | 110,043 | 6,861 (6.2) | | 0.77 (0.75-0.80) | 0.83 (0.70-1.00) |
| **Multiple gestation** |  | | | | | | | | |
| No | 731,068 | 52,557 (7.2) | Ref. | Ref. | 731,066 | 53,539 (7.3) | | Ref. | Ref. |
| Yes | 1,686 | 195 (11.6) | 1.68 (1.46-1.93) | 0.82 (0.26-2.56) | 1,686 | 176 (10.4) | | 1.50 (1.29-1.74) | 0.35 (0.05-2.47) |
| **Preterm labor** |  |  |  |  |  |  | |  |  |
| **No** | 664,590 | 46,306 (7.0) | Ref. | Ref. | 664,589 | 47,623 (7.2) | | Ref. | Ref. |
| **Yes** | 68,164 | 6,446 (9.5) | 1.42 (1.39-1.46) | 1.03 (0.89-1.18) | 68,163 | 6,092 (8.9) | | 1.31 (1.28-1.35) | 1.36 (1.08-1.71) |
| **PROM** |  |  |  |  |  |  | |  |  |
| **No** | 710,788 | 50,301 (7.1) | Ref. | Ref. | 710,786 | 51,465 (7.2) | | Ref. | Ref. |
| **Yes** | 21,966 | 2,451 (11.2) | 1.67 (1.60-1.74) | 1.41 (1.14-1.74) | 21,966 | 2,250 (10.2 | | 1.50 (1.44-1.57) | 0.94 (0.73-1.20) |
| **Gestational diabetes** |  |  |  |  |  |  | |  |  |
| **No** | 654,604 | 46,722 (7.1) | Ref. | Ref. | 654,602 | 47,780 (7.3) | | Ref. | Ref. |
| **Yes** | 78,150 | 6,030 (7.7) | 1.08 (1.06-1.11) | 1.10 (0.98-1.25) | 78,150 | 5,935 (7.6) | | 1.04 (1.02-1.07) | 0.93 (0.82-1.07) |
| **Gestational hypertension** |  |  |  |  |  |  | |  |  |
| **No** | 723,100 | 51,839 (7.2) | Ref. | Ref. | 723,098 | 52,827 (7.3)) | | Ref. | Ref. |
| **Yes** | 9,654 | 913 (9.5 | 1.34 (1.25-1.43) | 0.96 (0.68-1.35) | 9,654 | 888 (9.2) | | 1.28 (1.19-1.36) | 1.23 (0.89-1.69) |
| **Smoking during pregnancy** |  |  |  |  |  |  | |  |  |
| **No** | 60,703 | 4,301 (7.1) | Ref. | Ref. | 60,703 | 4,246 (7.0) | | Ref. | Ref. |
| **Yes** | 515 | 49 (9.5) | 1.44 (1.09-1.91) | 1.49 (1.02-2.18) | 515 | 48 (9.3) | | 1.44 (1.08-1.91) | 1.32 (0.87-1.99) |
| Abbreviations: NI, Neurodevelopmental impairments; FT, Full term; PT, Preterm; MLPT, Moderate-to-late preterm; VPT, Very-preterm; PROM, Premature rupture of membranes. | | | | | | |  |  |  |
| ^a^Crude. | | | | | | |  |  |  |
| ^b^Adjusted for gestational age groups, birth year, sex, cesarean section delivery, birth weight, abnormality of growth, history of breastfeeding, maternal age, socioeconomic status, multiple gestation, preterm labor, PROM, gestational diabetes, gestational hypertension, and smoking during pregnancy. | | | | | | |  |  |  |
| ^c^Classified by the criteria for normal, low, and high birth weight by WHO recommendation [20]. | | | | | | |  |  |  |
| ^d^Classified as normal (having both normal weight and height) and abnormal (either abnormal weight or abnormal height) based on the weight and height data measured at 4th screening following the growth curve suggested by Korean National Growth Charts for Children and Adolescents [21]. | | | | | | |  |  |  |
| ^e^National Health Insurance Premium quintiles (highest to lowest from 5 to 1). | | | | | | |  |  |  |
